# Content and Face Validation of a Novel, Interactive Nutrition Specific Physical Exam Competency Tool (INSPECT) to Evaluate Registered Dietitians’ Competence: A Delphi Consensus from the United States

**DOI:** 10.3390/healthcare9091225

**Published:** 2021-09-17

**Authors:** Sunitha Zechariah, Jennifer L. Waller, Gianluca De Leo, Judith Stallings, Ashley J. Gess, Leigh Lehman

**Affiliations:** 1 College of Allied Health Sciences, Augusta University, Augusta, GA 30912, USA; gdeleo@augusta.edu (G.D.L.); jstallin@augusta.edu (J.S.); 2 Medical College of Georgia, Augusta University, Augusta, GA 30912, USA; jwaller@augusta.edu; 3 College of Education, Augusta University, Augusta, GA 30912, USA; AGESS@augusta.edu; 4 School of Occupational Therapy, Brenau University, Gainesville, GA 30501, USA; llehman@brenau.edu

**Keywords:** Delphi consensus, nutrition-focused physical exam, content validity, face validity, registered dietitian nutritionists, competency

## Abstract

The nutrition-focused physical examination (NFPE) is an integral component of nutrition assessment performed by registered dietitian nutritionists (RDNs) to determine signs of malnutrition and other nutrition-related complications. Increased use of this essential skill among RDNs and the transformation of dietetics education to a competency-based model in the near future calls for appropriately validated tools to measure RDNs’ NFPE competence. To fill the need for a validated competency tool, this study developed an Interactive Nutrition-Specific Physical Exam Competency Tool (INSPECT) utilizing the initial 70 items identified in the first phase of the study. The second phase of this study aimed to test the preliminary version of the INSPECT for content and face validity. An expert panel of 17 members provided consensus recommendations through the Delphi process. Internal consistency of the consensus was measured with Cronbach’s alpha (α) and α of ≥0.70 was defined as acceptable a priori. Inter-rater agreement among the expert panel was determined using the intraclass correlation coefficient (ICC) and an a priori ICC of 0.75 to 0.9 was established as good and >0.9 as excellent agreement. The results showed acceptable face validity (α = 0.71) and excellent content validity for the INSPECT, with an internal consistency of α = 0.97 in the first round and α = 0.96 in the second round. The inter-rater agreement was also excellent with ICC = 0.95 for each of the Delphi rounds. A total of 52 items were retained from the preliminary version of the INSPECT. Open feedback from the experts allowed for the consolidation of 11 similar items for better scoring and evaluation and thus, a total of 41 items were included in the final version of the INSPECT. The final version of the INSPECT is currently being studied in real-life, multi-site clinical settings among practicing RDNs to examine construct validity, reliability, and item-level psychometric properties. Ultimately, the validated INSPECT will be available for the competency evaluation of RDNs practicing in clinical settings.

## 1. Introduction

Nutrition-focused physical examination (NFPE) is a systematic head-to-toe assessment of the physical and functional abilities of patients to determine their nutritional status and to verify the presence of any nutrient deficiencies or excesses [1]. Registered dietitian nutritionists (RDNs) provide medical nutrition therapy (MNT) to hospitalized patients to diagnose and treat nutrition related problems. MNT allows RDNs to provide individualized, evidence-based, nutrition therapy service for patients who require nutritional intervention [2]. As part of the MNT, RDNs conduct nutrition assessments, identify nutrition diagnoses, establish goals, develop therapeutic interventions and care plans for the patients. The first step in the MNT is a comprehensive nutrition assessment conducted by the RDNs. A nutrition assessment involves collecting, examining and inferring a variety of patient information including anthropometrics, biochemical parameters, clinical evaluation and diet history to determine the cause and extent of the patients’ nutrition problems [2,3]. An integral component of the clinical evaluation part of the nutrition assessment is a hands-on head-to-toe physical examination that enables the RDNs to gather accurate information to identify nutritionally relevant signs and deficiencies. Specifically, NFPE is a valuable tool for the RDNs to visually inspect and palpate for areas of muscle wasting, determine losses in subcutaneous fat, identify areas of fluid accumulation, and the presence of reduced grip strength. The results from NFPE are then consolidated with other pertinent information to assess the patients’ nutritional status and any existing nutrient insufficiencies. Despite NFPE being a useful tool, many RDNs are not comfortable performing the physical exam due to limited technical training, lack of consistent practice, time constraints, and reluctance to physically touch the patients [4,5]. Attempts are being made to train RDNs to employ NFPE in their routine practice, however, wide variation in skill and comfort level in performing NFPE has been reported among RDNs [4,6].

A few years ago, NFPE was added as an essential skill for RDNs currently in clinical practice and for dietetic students as the dietetics education is on the trajectory to become a competency-based education model in the future [7,8]. Several health professions including medicine, nursing, and pharmacy have embraced competency-based education as an effective model to prepare future healthcare professionals to provide reliable, effective, safe, and patient-centered care [9,10]. Healthcare professionals including RDNs not only must acquire knowledge but also should have the capability to translate the knowledge into meaningful practice, communicate and collaborate effectively with team members, treat patients with utmost respect and compassion, uphold professional ethics, and dedicate time to be lifelong learners [9]. In order to authentically prepare students for the morphing challenges in healthcare practice, there is a pressing need for health profession educators to expand upon the traditional didactic instruction and antiquated multiple-choice assessments [9]. The future educational system needs to be a model that facilitates skill acquisition, meets strategic competencies, impels continuous learning, and activates sustained retention of competence [11,12]. As health education evolves to adopt these qualities through competency-based education models, healthcare educators are proposing evaluation tools to rigorously test and verify the mastery of competence, going beyond the walls of academic institutions to throughout the entire career of healthcare providers [9,13,14].

The Academy of Nutrition and Dietetics (the Academy), a professional nutrition and dietetics organization for RDNs has published the Scope of Practice, Standards of Practice (SOP) and Standards of Professional Performance (SOPP) [15] and the Accreditation Council for Education in Nutrition and Dietetics (ACEND), the accrediting body for dietetics education has stipulated the educational requirements [7,8], providing the framework for demonstration of competence beginning from the time of dietetic education to throughout the RDNs’ professional practice, particularly for those practicing in patient-care settings. In addition, regulatory agencies overseeing hospital accreditation such as the Centers for Medicare and Medicaid (CMS) and The Joint Commission (TJC) require hospitals to ensure that the “staff are competent to perform their responsibilities” and call for annual competence assessment of all staff (TJC Standard HR.01.06.01) [16]. TJC expects hospitals to verify the initial and ongoing competency of their healthcare providers using qualified individuals and tools that incorporate performance validation components [16].

Evaluating the RDNs’ initial and ongoing hands-on NFPE competence needs to be part of a comprehensive competency assessment plan to gauge their ability to accurately assess patients’ nutritional status, diagnose protein-calorie malnutrition and other nutrient deficiencies, to ensure overall safe and effective patient care, to perform within the scope and professional standards of practice set forth by the Academy, and to satisfy the verification of skills required by healthcare accreditation and other regulatory agencies [6,15,17,18]. A review article on physical assessment skills for dietetics practice has called for a common set of agreed-upon NFPE terms and competencies for clinical practice [6]. Although the need for competency tools to evaluate NFPE skills was identified decades ago, validity and reliability tested tools are severely limited. Hence, the need arose to design the INSPECT [19] and scientifically validate it to evaluate RDNs’ NFPE competence.

In the first phase of the study, NFPE tool items were generated by utilizing the expertise of content and practice experts through technology-based focus group discussions [19]. The generated items were then utilized to develop a preliminary version of the tool, the Interactive Nutrition-Specific Physical Exam Competency Tool (INSPECT) [19]. The next step is to validate the INSPECT beginning with the preliminary types of validity. Two commonly used initial types of validity in tool development are face and content validity. Face validity examines whether the items in the tool appear to be appropriate, reasonable, unambiguous, and clear [20,21,22]. Content validity measures whether the items on the tool are relevant and representative of the target construct [21,23]. Face and content validity are typically based on expert opinions. Although, these types of validity are highly subjective in nature, examining the face and content validity is a critical step in the development process of new tools as they ensure that the tool design and construct are pertinent prior to investigating for reliability and more rigorous types of validity studies [24].

Various types of data collection methodologies have been utilized to examine face and content validity in tool development such as interviews and face to face discussions [25,26]. While these methodologies are useful, they may hinder free expression of participants’ views in a non-threating environment. This study employed the Delphi technique, as this methodology is an iterative, multistage process that allows experts to independently review the information and provide their opinion until a consensus is reached [27,28]. The Delphi process is structured to maintain anonymity between participants in order to avoid group thinking and influence by the other participants [29]. The Delphi method is conducted in multiple rounds allowing the participants numerous occasions to convey their opinion. At the end of each round, deidentified, collated responses are shared with the expert panel, allowing them the opportunity to reevaluate their views as needed. This approach facilitates group consensus without the experts being subjected to undue pressure and influence from other panel members [28]. Hence, the second phase of this study aimed at measuring the face and content validity of the preliminary version of the INSPECT, utilizing NFPE content experts through the Delphi methodology.

## 2. Methods

### 2.1. Item Generation and Initial Tool Design

Using the items generated from the expert focus group discussions in the first phase of the study, a preliminary version of the INSPECT that incorporated all areas of physical assessment was developed. The methodology of item generation and identification of NFPE components has been described in detail in a previous publication [19]. The competency tool, the INSPECT was developed containing a total of 70 items identified from the expert focus groups. The tool items were categorized into 13 subsets based on a head-to-toe sequence. Each subset consisted of a varying number of items ranging from 3 to 13 depending on the exam area. For example, the eye exam subset had 4 items while the upper extremities exam had 13 items. Each NFPE item under each subset was provided with performance indicators explaining how the exam should be conducted on the patient along with a scoring scale.

The INSPECT was designed using Microsoft Excel^TM^ (2016) with formulas embedded to calculate scores automatically. The tool calculates scores for each subset based on whether an item is rated as ‘complete’ with a score of ‘1’ or as ‘incomplete’ with a score of ‘0’. For any item that does not apply to the patient, ‘not applicable’ or ‘NA’ is assigned. The INSPECT calculates scores for each subset by adding all the items that score ‘1’ and deducting the items that are not applicable (‘NA’). The INSPECT also computes an overall NFPE score, an overall percentage, overall total points possible, and overall total points missed.

### 2.2. Participant Selection

In this second phase of the study, content experts practicing within the dietetics field were identified depending on their clinical experience, background knowledge, and practice in NFPE. Inclusion criteria for the experts included a minimum of 2 years of clinical experience and 2 years of performing NFPE on adult patients. Twenty NFPE experts from around the United States were identified and invited to participate in the study, including the experts who participated in the focus group discussions during the first phase of the study. An email invitation was sent to each of the expert RDNs along with a description of the study and a web-based consent form. The institutional review board of Augusta University reviewed the study and determined it to be exempt from full board review (#1643906-1).

### 2.3. Delphi Methodology

Utilizing the Delphi technique, the expert panel was asked to review and rate the INSPECT for face and content validity. The rating instructions and rating scales for face and content validity, a demographic questionnaire, and a copy of the INSPECT were distributed via encrypted email for faster dissemination [30]. Two rounds of Delphi were conducted, and additional rounds would have been considered if there had been substantial discrepancy among the experts [30,31]. Experts were given two weeks to rate the INSPECT during each round. Email reminders were sent after one week to the experts who failed to respond to the initial email. Each of the experts was assigned a code for identification and reference purposes.

### 2.4. Face Validity of the INSPECT

Evidence for face validity of the INSPECT was examined by the expert panel using 8 dichotomous items with options of ‘Clear’ and ‘Not Clear’. The 8-item face validity scale was developed by the authors to measure the overall appearance of the INSPECT. The 8-item scale comprised of: (1) clarity of instructions to complete the tool, (2) organization of the tool in a head-to-toe sequence, (3) clarity and ease of tool scoring system, (4) clarity of items within each subset, (5) consistency in language style, (6) accuracy in the scoring of the tool subsets, (7) accuracy in overall scoring of the tool, and (8) good layout of the tool [21,32,33]. To calculate face validity, a score of ‘1’ was assigned to “Clear” and a score of ‘0’ was assigned to “Not Clear” for each of the ratings and the mean score was calculated for each item [34]. In addition to the 8-item scale, experts were asked to describe what the tool intended to measure to validate the degree to which the INSPECT appears to be related to the NFPE construct.

### 2.5. Content Validity of the INSPECT

Utilizing the Delphi technique, the content experts were asked to review anonymously and independently each of the INSPECT item using a 5-point Likert scale [30,35]. The Likert scale was set as 1 = not important, 2 = sometimes important, 3 = important, 4 = very important, and 5 = essential [30,35,36]. Content experts were asked to examine the items for representativeness of the breadth of the construct [32]. The first-round consensus level was set a priori at >50% agreement for those items that received a score of 4 or greater on the 5-point Likert scale [30,37,38,39]. For example, if >50% of the experts scored an item at a level of ‘4’ or ‘5’, then it was considered that more than 50% of the expert panel agreed on the item to be ’very important‘ or ’essential‘ and therefore it was retained. All items that received consensus from the expert panel in the first Delphi round were deemed as essential components and were retained in the INSPECT. The retained items from the first round were made available in the second Delphi round, however, they were not reexamined in the second round. Those items that were scored ‘1’ by the experts, that is ‘not at all important’ were considered as not relevant. Any item that received a score of ‘1’ and had a 100% agreement among the expert raters was set to be excluded from the INSPECT. The remaining items were reevaluated by the expert panel in the second round of Delphi.

The second round of Delphi consensus was set a priori at >50% agreement for those items that received a score of ≥3 on the 5-point Likert scale [30,37,38,39]. The overall panel results from the first round (lowest and highest ratings), expert’s individual rating compared to the panels’ median rating, whether consensus was reached for an item, and an anonymized summary of all qualitative comments were provided to each panel member for the second round of ratings [29,36,37]. The summarized data provided to the panelists informed them of their position in relation to the entire panel and allowed the panelists an opportunity to change their judgment if desired. The consensus achieved by these expert panelists was used to modify the INSPECT to create a second version. The identity of individual panelists and their personal ratings were never shared with the panel members and were kept confidential throughout the study [36]. An a priori decision was made to conduct the third round of Delphi only if at least 50% of the total tool items (35 items) were not agreed upon by the experts.

### 2.6. Open Comments of the INSPECT

In addition to the face and content validity rating scales, experts were invited to provide suggestions on any aspect of the INSPECT to enhance the design, content, and scoring of the tool [29,35]. Free text space for each item of the INSPECT and an overall comment box were provided for the experts to add additional comments as needed [29,36]. Experts had the opportunity to provide comments for each of the Delphi rounds.

### 2.7. Statistical Analysis

Statistical analysis was performed using the SPSS version 25 (IBM SPSS, Inc., Armonk, NY, USA). Consensus or internal consistency between experts was determined using Cronbach’s alpha and was defined a priori at an acceptable level of at least 0.70 for face and content validity [32]. A two-way mixed-effects intraclass correlation coefficient (ICC) model with the panelists considered a random effect, was used to determine overall inter-rater agreement. ICC was selected to determine the overall agreement among the expert panelist as ICC is the preferred measure for 5-point Likert scale type of data, primarily in Delphi studies in the healthcare field [29,40,41,42,43,44]. An ICC between 0.75 and 0.90 was considered as good and >0.9 as excellent overall agreement among the expert panel in each of the Delphi rounds. An ICC between 0.50 and 0.75 was considered as an acceptable inter-rater agreement for face validity. ICC cut-off points were determined using Koo and Li’s interpretations [32,45]. Demographic information of the content experts such as their age, gender, highest degree attained, job title, years of clinical practice, years of NFPE practice, and practice location was analyzed using appropriate descriptive statistics.

## 3. Results

**Figure 1:**
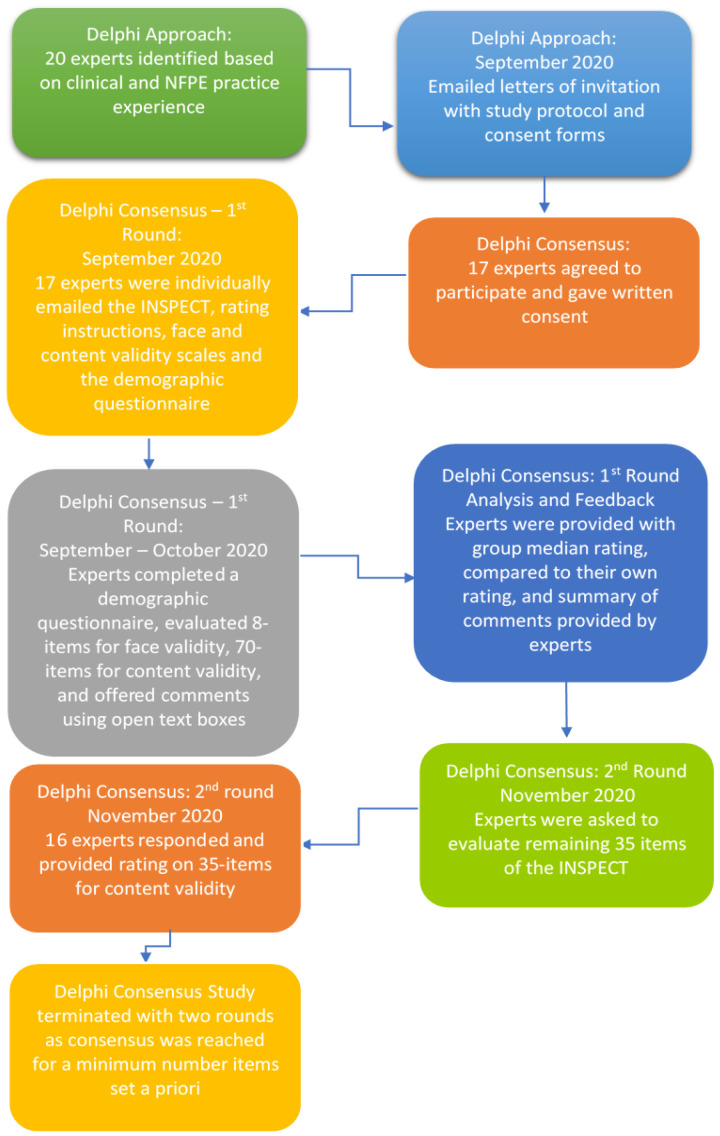
The flow chart of Delphi consensus for the INSPECT.

Seventeen of the 20 invited experts agreed to participate by providing written consent, resulting in an 85% response rate. The response rate was assumed appropriate as a sample size of 15–30 panelists is considered adequate for the Delphi studies [46]. All experts (*n* = 17, 100%) took part in the first round of Delphi while one expert did not respond in the second round (*n* = 16) resulting in a 94% participation rate. Figure 1 shows the flow chart of the Delphi process for the face and content validation of the INSPECT.

The demographic characteristics of content experts as outlined in Table 1 showed a median age of 48.5 years (range = 36.2–60.7), a median clinical dietetic experience of 13 years (range = 6.5–31.5), and a median NFPE practice experience of 6 years (range = 4.5–21.5). All participants identified themselves as White, non-Hispanic females. A majority of the participants had completed graduate degrees, were employed as clinical dietitians or clinical dietitian specialists, and worked in acute care hospitals.

### 3.1. Face Validity of the INSPECT

All participants rated the 8-item face validity scale during the first round of Delphi. Fourteen content experts (82.4%) found the overall layout of the INSPECT to be good and the subsets were set to score accurately. Fifteen experts (88.2%) found the total scoring system clear and easy to use and the overall tool score calculations were accurate. Three experts (17.6%) suggested improving the INSPECT’s binary scoring (complete/incomplete) to include scoring for partial completion of items in order to assess RDNs’ ongoing progress of NFPE skill development. Thirteen participants (76.5%) concurred that the INSPECT was organized in a logical, head-to-toe assessment. Four experts (23.5%) did not favor the head-to-toe sequence and instead preferred to begin with the hand exam. Eleven experts (64.7%) agreed that the instructions to complete the tool were clear and the items within the subsets were clearly written. Six participants (35.3%) provided suggestions to improve the language within the tool in the open comment section. The face validity ratings for each of the items and the 95% confidence intervals are depicted in Table 2. The overall consensus or internal consistency of the expert group for face validity was found to be acceptable with a Cronbach’s alpha of 0.71 [32]. The inter-rater agreement for face validity was also found to be acceptable with an ICC of 0.71 [45].

For the question on what the experts believed that the INSPECT was purported to measure, 94% (*n* = 16) of the experts responded that the tool intended to measure the competency of RDNs’ NFPE performance. One participant had a missing response to this question.

### 3.2. Content Validity of the INSPECT

All experts assessed content validity on the 70 items of the INSPECT using the 5-point Likert scale in the first round of Delphi. Over 50% of the expert panel assigned a score of 4 or greater for 35 items on the INSPECT, indicating that these items are either ‘very important’ or ‘essential’. The overall consensus or internal consistency of the expert group for content validity in Delphi round one was found to be excellent with a Cronbach’s alpha of 0.97 [32]. The inter-rater agreement measured by ICC was 0.95, showing excellent agreement among the expert panel for round one [45]. All 35 items were retained in the INSPECT and were not reassessed during round two of Delphi. No items were rejected in the first round since none of the items were given a score of ‘1’ by 100% of the expert panel.

Round two of the Delphi began with the remaining 35 items of the INSPECT. During round two, 17 items received a score of ≥3 on the 5-point Likert scale from more than 50% of the expert panel. These 17 items were retained as ‘Important’ components of the INSPECT. The overall consensus of the expert group for content validity in Delphi round two was found to be excellent with a Cronbach’s alpha of 0.96 [32]. The inter-rater agreement measured by ICC was found to be 0.95 showing excellent agreement among the expert group for the 35 items reassessed during round two of Delphi [45].

A total of 52 items were included in the second version of the INSPECT based on the expert consensus during the two Delphi rounds. Since the Delphi consensus was reached for ≥50% of total items on the INSPECT, a third round was not considered necessary, and the study was terminated after two rounds. The remaining 17 items from round two were rejected as ‘Not Important’ and were eliminated from the INSPECT. Table 3 presents the initial 70 items of the INSPECT evaluated by the experts in each of the Delphi rounds, the medians, the upper and lower quartiles for each round, and the decision to retain, reassess or reject the items for each round.

Experts provided additional feedback using the open comment section to enhance the design, scoring, and language of items on the tool. All appropriate suggestions were accepted and the INSPECT was revised accordingly. For example, a few experts suggested expanding the scoring from a binary system to include scores for partial completion of each item in order to detect progress in NFPE skill development as RDNs gain experience with practice and training. Considering the suggestion from the experts, and after additional literature review [18], the INSPECT was redesigned to include a scale of ‘complete’, ‘partially complete’ and ‘incomplete’. The ‘not applicable’ option was retained in the tool. Some experts proposed to alter the head-to-toe sequence of the tool to begin with the hand exam. However, as a majority of the experts favored the head-to-toe exam format and further exploration of the literature revealed this sequence to be the preferred choice in physical exams [17,47,48], and hence, the head-to-toe sequencing was retained in the INSPECT. Experts also offered recommendations to improve the language of some of the items on the INSPECT. All suitable suggestions were incorporated to enhance the clarity of the items. For example, two experts recommended adding clarification on the patient being alert and oriented to obtain verbal consent, which was added to the item in the INSPECT. Similarly, a few experts felt that the term ‘ill fitting’ should be added about dentures and it was incorporated in the INSPECT. Two experts wanted an explanation for the need to have ‘not applicable’ as a scoring option and hence a brief description was added to the scoring scale of the INSPECT.

## 4. Discussion

This study, as far as the authors know, is the first attempt to achieve consensus among actively practicing expert RDNs from across the United States to measure the face and content validity of a core set of NFPE components needed to adequately evaluate RDNs’ NFPE competence. The preliminary version of the INSPECT is designed to offer a validated measurement of the RDNs NFPE competence, which in turn will allow the RDNs to accurately assess malnutrition, micronutrient imbalances and other nutrition related concerns in their patients. Furthermore, consistent and accurate measurement of the RDNs’ NFPE competence utilizing appropriately developed and validated tools such as the INSPECT is crucial as it enables the clinical supervisors to verify the RDNs’ mastery of competence and to ascertain the instructional training needs for skill-building. The Delphi methodology was utilized for the purpose of achieving consensus among the expert RDN panel. This methodology was appropriate as it allowed each participant to anonymously and independently formulate their own opinion and rate the INSPECT without undue influence of other panel members [40,49]. In addition, the Delphi technique offers greater advantages for reaching consensus compared to other methods such as round table discussions, face-to-face meetings or task force workgroups as these methods may introduce bias by domineering individuals and curtail free expression of opinions by all panel members [29,40,50,51]. Moreover, the Delphi method is widely accepted in the healthcare types of research for obtaining systematic consensus as it offers unbiased, independent expert views, which are highly preferred for studies of explorative nature [27,30,52]. The Delphi methodology also allowed for consensus from a wide reach of clinically and geographically diverse experts in a shorter time frame without the need for travel [29]. This method also facilitated an inexpensive, electronic distribution of the INSPECT along with the rating scales and allowed for faster return of responses.

The overall consensus by the expert panel showed acceptable face validation in terms of the tool’s readability, consistency of style, clarity of language, sequencing of subsets, scoring of subsets, the overall layout of the tool and that the INSPECT appeared to be related to the NFPE construct [21]. A resounding majority of the experts also concurred that the INSPECT intends to measure the RDNs’ competency in performing NFPE. Content validation by the expert panel revealed agreement for 52 items out of the initial 70 items during the two Delphi rounds. As endorsed by experts during the first phase of this study [19], the Delphi panel, in general, had a high-level consensus on items related to macronutrient deficiencies including muscle atrophy, subcutaneous fat loss, and fluid retention. Several previous studies, reports and reviews confirm this finding and concur with the expert consensus [6,17,53,54]. Agreement among the panel was low for testing functional grip strength during the first Delphi round and only the subjective measurement of handgrip reached consensus in the second round. A few open comments from the experts indicated that the reasons for low consensus for the objective measure of grip strength using a hand dynamometer were due to the unavailability of the equipment in their facilities, difficulty using the dynamometer in clinical settings, and lack of training on the standardized measurement process. The decreased usage of handgrip strength as a measure was previously confirmed by a nationwide practice survey among clinicians in 2012 [55]. Although the practicality of accessing and using a hand dynamometer to test patients’ grip strength in acute care facilities is challenging, RDNs should explore the feasibility of collaborating with physical and occupational therapists to complete this measurement as these therapists usually have access to hand dynamometers. Additionally, RDNs could utilize alternate parameters such as exploring the patients’ ability to perform activities of daily living, capacity to tolerate physical therapy, and the ability to wean from mechanical ventilation to determine the functional status of the patients [54].

Interestingly, there was mixed consensus among the expert panel for items related to identifying micronutrient deficiencies. Of the 31 items examining micronutrient deficiencies in the INSPECT, only 9 items received consensus in Delphi round one and 15 more items were agreed upon in Delphi round two. In total, 24 items related to micronutrient deficiencies were retained based on expert consensus. The World Health Organization specifies deficiencies in vitamins and minerals as micronutrient-related malnutrition [56], which can develop and become apparent during malnutrition caused by acute or chronic disease states as well as during starvation [57,58]. Since RDNs have the knowledge and training to recognize deficiencies related to vitamins and minerals, it would be prudent to employ NFPE as a tool to gather micronutrient deficiency related information particularly due to the rise in the aging population [57,59,60]. Specific training focused on micronutrient deficiency exams should be made available to the RDNs, which will aid them in performing these exams with confidence and accuracy.

The experts did not arrive at a consensus for any of the items related to the abdominal exam. Although consensus improved from round one to round two, less than 50% of the experts agreed that items related to the abdominal exam were essential in the tool. Hence, all 3 items related to the abdominal exam were rejected at the end of the two Delphi rounds. This finding was similar to the results from the first phase of the study, where only 2 of the 7 focus group experts agreed that abdominal exam is a necessary skill for the RDNs [19]. Open feedback from the Delphi rounds revealed that the expert RDNs relied on nurses to conduct abdominal exams and, in particular, auscultation of bowel sounds. Surprisingly, a Delphi study exploring the core skills for nursing physical assessment found that the nurses did not consider abdominal exams as a core nursing skill in the acute care setting [35]. Therefore, as RDNs continue to advance their NFPE skills and contribute as part of the interdisciplinary team, this may be a new opportunity for the RDNs to assume the responsibility of auscultation of bowel sounds. Meanwhile, RDNs, nurses and other interdisciplinary members should continue to collaborate and to communicate their physical exam findings to the team for appropriate treatment of the patients.

Similarly, less than 50% of the expert panel agreed on items related to the basic swallow examination and very minimal change in expert consensus was noted between Delphi rounds one and two. It is well established that dysphagia is a major etiology of malnutrition in older adults [61]. Given the rise in the older adult population of ≥65 years, which is predicted to double to 1.5 billion by 2050 [62], swallowing assessment in older adults is a crucial part of malnutrition diagnosis. The rapid increase in the older adult population may mean there is a likelihood of increased number of older adults at risk for malnutrition [61]. In the United States, speech therapists are the professionals licensed to evaluate swallowing disorders. Potentially, RDNs could participate in conducting basic swallow exam on patients to support the speech therapists as it is within the scope of RDNs practice [15] and it has been added to the just released version of 2022 ACEND standards for dietetics students [63].

All the items related to the ‘preparation and initial steps’ prior to NFPE and ‘bedside manner and etiquette’ while performing NFPE reached high consensus during the first Delphi round. In total, 52 items were retained in the INSPECT. While designing the INSPECT with the finalized items, feedback from the experts was thoroughly scrutinized and incorporated where appropriate. One of the main comments from the experts’ feedback indicated that inspection of individual areas of micronutrient deficiencies by an RDN would be difficult to score by an observer during a competency evaluation. For example, when the RDN is inspecting the nails, it would be difficult to determine if the RDN is inspecting for nail color or koilonychia or Beau’s lines or splinter hemorrhage etc., unless the RDN verbalizes their observation, which is not feasible or realistic in a real-life clinical setting. Based on this feedback, all items related to a specific area of inspection were combined. For instance, items related to nail inspection were pooled to become one item on the INSPECT. Similarly, items related to the eye exam, and the hair exam were pooled where possible to allow for proper observation and scoring during a competency evaluation. Thus, the final version of the INSPECT resulted in 41 items using which an interactive tool has been created to provide immediate scores upon completion of an NFPE competency evaluation.

The current study has established the content and face validation for the INSPECT, however, it is important to conduct further studies to ascertain the reliability and other types of validity measures through direct field-testing among practicing RDNs [36,64]. While content and face validity are essential to measure if the tool reflects the construct and has practical utility, they are preliminary types of validity [21,22]. Therefore, further rigorous validation measures should be investigated in clinical settings. Furthermore, while expert consensus is an excellent method to determine tool items, further research is required in a real-life clinical setting to refine the INSPECT [35,65]. Hence, the final version of the INSPECT is currently being field-tested nationwide among RDNs in clinical practice to examine the reliability, other types of validity, and the psychometric properties. This further investigation will assist in further validating the INSPECT for routine use among RDNs in clinical practice.

## 5. Strengths and Limitations

Recognizing the need for a validated competency tool to evaluate the NFPE performance of RDNs, this study designed the INSPECT and conducted preliminary validation studies with expert consensus using the Delphi methodology. To the knowledge of the authors, this study is the first to employ the Delphi technique among a meticulously selected expert raters to validate a NFPE competency tool. Experts were selected through purposive sampling with predefined inclusion criteria. Expert panelists were chosen from a variety of practice settings with a wide range of clinical and NFPE practice experience and were from diverse geographical locations throughout the United States. Utilizing experts practicing in the field with a wide range of experience to validate the INSPECT is a major strength of this study as experts bring not only the theoretical knowledge, but also the practical aspects of performing NFPE on the patients. During the Delphi rounds, the panelists were able to provide anonymous and independent rating of the INSPECT and were able to freely express and/or modify their opinions without the influence of the other participants, which is an additional strength of this study. The minimal dropout rate between Delphi rounds rendering a sufficient sample size for both rounds is an added strength. Another strength is that all measurements of consensus for face and content validity were established a priori and open feedback from the experts was given equal importance to improve the design and functionality of the INSPECT.

An inherent limitation of this study is that it is based on a limited number of experts’ opinions who were only recruited from the United States. Expanding this study to include a larger number of local and international experts would be beneficial to ensure depth and diversity arising from their responses. Additionally, increasing the number of Delphi rounds may prove valuable to improve consensus of items that were rejected after two rounds. However, respondent fatigue should be taken into consideration with excessive iterative rounds. The cut off points selected could also be tightened in future studies to ensure absolute consensus among the expert panel.

## 6. Conclusions

This is an ongoing study to design and rigorously validate the INSPECT to evaluate RDNs’ competence in performing NFPE to ensure accurate diagnoses of malnutrition and other nutrition-related problems. Items generated in the first phase of the study were used to design the INSPECT, and this Delphi study explored the expert consensus resulting in excellent inter-rater reliability and internal consistency for content validation and an acceptable level of face validation. The final version of the INSPECT is currently being evaluated in real-life, multi-site clinical settings among practicing RDNs to establish additional evidence supporting the INSPECT’s construct validity, reliability, acceptability, and feasibility. Ultimately, the validated INSPECT will be available to evaluate the competence of RDNs practicing in clinical settings. As the RDNs prepare for a future with a competency-based education model, appropriately developed and validated tools such as the INSPECT will become crucial to assess RDNs’ competence. Competent RDNs are indispensable as they provide collaborative, interdisciplinary team-based care for all patients, especially for the increasing, vulnerable aging population.

## Figures and Tables

**Table 1:** Demographic Characteristics of Content Experts.

Variable (*n* = 17)	Median (Q1–Q3) ^	*n*	%
Age (years) *	48.5 (36.2–60.7)		
**Gender**			
Females		17	100
**Ethnic Background**			
White, non-Hispanic		17	100
**Highest Degree Earned**			
Bachelors		5	29.4
Masters		7	41.2
Doctoral		5	29.4
**Primary Job Role**			
Clinical Dietitian/Specialist		7	41.1
Clinical Nutrition Manager/Lead Dietitian		6	35.3
Educator/Researchers		4	23.5
**Primary Work Location**			
Inpatient Hospital		11	64.7
Outpatient Clinic		2	11.8
Academic Institutions		4	23.5
**Years of Practice as Clinical Dietitian**	13 (6.5–31.5)		
**Years of Experience in Performing NFPE**	6 (4.5–21.5)		

* Age is reported for *n* = 16 due to one missing data. ^ Q1—Lower Quartile, Q3—Upper Quartile.

**Table 2:** Face Validity Rating of the INSPECT.

Face Validity Items (*n* = 17)	Clear	Not Clear	95% Confidence Interval for Clear	
	%	%	LB *	UB ^
Instructions on completing the tool are clear and easy to understand	64.7	35.3	39	65
The tool is organized in a logical way, following a head to toe assessment	76.5	23.5	54	76
Tool scoring system is clear and easy to use	88.2	11.8	71	88
Each item within each subset is clearly written	64.7	35.3	39	65
Each item has a consistent style in language	70.6	29.4	46	71
Subset scores calculate accurately taking into account ‘not-applicable’ items	82.4	17.6	62	82
Overall tool score calculates accurately	88.2	11.8	71	88
The layout of the tool is good	82.4	17.6	62	82

* LB—Lower Bound, ^ UB—Upper Bound.

**Table 3:** Delphi Consensus on the Content Validity of the INSPECT.

		Delphi Round 1 (*n* = 17) (Items = 70)					Delphi Round 2 (*n* = 16) (Items = 35)				
INSPECT Categories	Items	Median	Quartiles		Agreement (>50% with ≥4)	Retain, Reject or Reassess	Median	Quartiles		Agreement (>50% with ≥3)	Retain, Reject or Reassess
			Q1 *	Q3 ^				Q1 *	Q3 ^		
Preparation & Initial Steps	Washes/Sanitizes Hands	5	5	5	94%	Retain					
	Utilizes PPE	5	5	5	94%	Retain					
	Introduces Self/Exam	5	5	5	94%	Retain					
	Verbal Consent	5	3	5	77%	Retain					
Head & Hair Exam	Asks Hair Changes	3	3	4	47%	Reassess	3	2	4	69%	Retain
	Inspects Dry/Dull Hair	3	3	4	44%	Reassess	3	2	4	53%	Retain
	Brittle Hair Pluckability	3	3	4	47%	Reassess	3	2	4	56%	Retain
	Seborrheic Dermatitis	3	2	4	36%	Reassess	3	2	4	50%	Reject
	Alopecia	3	2	4	24%	Reassess	2	2	3	44%	Reject
Face Exam	Flakiness -Nasolabial area	4	3	4	65%	Retain					
	Facial Movements	3	2	4	35%	Reassess	2	2	3	31%	Reject
	Temporal Muscles	5	4	5	94%	Retain					
	Buccal Fad Pads	5	3	5	88%	Retain					
	Temporomandibular Joint Range of Motion	2	2	4	29%	Reassess	2	1	3	50%	Reject
Eye Exam	Conjunctivae Color	4	3	5	59%	Retain					
	Bitots Spots	3	3	4	47%	Reassess	3	2	4	56%	Retain
	Nystagmus	3	2	4	35%	Reassess	2	1	4	25%	Reject
	Orbital Fat Pads	5	4	5	77%	Retain					
Mouth & Oral Cavity Exam	Dentures	2	2	4	41%	Reassess	4	1	4	75%	Retain
	Perioral Areas	4	3	5	53%	Retain					
	Angular Stomatitis/Cheilosis	4	3	5	59%	Retain					
	Oral Ulcer/Lesions	3	2	4	35%	Reassess	4	2	4	69%	Retain
	Inspects Gums/Teeth	4	3	5	53%	Retain					
	Inspects Buccal Mucosa	3	2	4	29%	Reassess	2	2	4	31%	Reject
	Inspects Tongue Filiform Papillary Atrophy	3	3	5	47%	Reassess	4	3	5	81%	Retain
	Magenta/Beefy Red Tongue	4	3	5	53%	Retain					
	Glossitis	4	3	4	53%	Retain					
	Inspects Uvula Midline Soft Palate Rising	2	2	4	29%	Reassess	2	1	3	25%	Reject
	Inspects Tongue Protrusion Movement	2	2	5	29%	Reassess	2	1	3	38%	Reject
Neck Exam	Inspects Swallow	2	2	5	29%	Reassess	2	1	3	31%	Reject
	Sternocleidomastoid Muscles Resistance	2	1	4	24%	Reassess	2	1	3	31%	Reject
	Trapezius Muscles Resistance	2	2	4	35%	Reassess	3	2	4	50%	Reject
	Inspects/Palpates Thyroid	2	1	2	6%	Reassess	2	1	2	6%	Reject
Clavicular/Thoracic Region Exam	Palpates Pectoralis	4	3	5	71%	Retain					
	Inspects/Palpates Deltoids	4	4	5	88%	Retain					
	Inspects Acromion Protrusion	5	4	5	82%	Retain					
	Inspects/Palpates Intercostal Muscles	4	3	5	71%	Retain					
	Palpates Muscles along Midaxillary Line	4	2	5	53%	Retain					
	Inspects Iliac Crest Prominence	4	3	5	53%	Retain					
Abdomen Exam	Palpates Abdomen	2	2	3	18%	Reassess	2	1	3	38%	Reject
	Percussion of Abdomen	2	2	3	18%	Reassess	2	1	3	38%	Reject
	Auscultation of Abdomen	2	2	2	18%	Reassess	2	1	3	25%	Reject
Back/Scapular Region Exam	Examine Back Skin	3	2	4	29%	Reassess	3	2	4	50%	Reject
	Inspects/Palpates Posterior Trapezius	4	3	4	65%	Retain					
	Inspects/Palpates Scapula	4	3	5	71%	Retain					
Upper Extremities Exam	Skin exam on upper & lower arm	3	3	5	47%	Reassess	3	2	4	69%	Retain
	Follicular Hyperkeratosis	3	2	4	41%	Reassess	3	2	4	69%	Retain
	Corkscrew Hair	3	2	4	29%	Reassess	3	2	4	69%	Retain
	Lanugo Hair	3	2	4	29%	Reassess	3	2	4	69%	Retain
	Palpates Triceps	4	3	5	71%	Retain					
	Inspects Nail Color	4	3	5	53%	Retain					
	Koilonychia	4	3	5	53%	Retain					
	Beau’s Lines	3	3	5	35%	Reassess	3	2	4	63%	Retain
	Splinter Hemorrhage	3	3	4	41%	Reassess	3	2	4	63%	Retain
	Clubbing Nails	3	3	4	47%	Reassess	3	2	4	63%	Retain
	Capillary Refill	3	2	4	41%	Reassess	2	2	4	44%	Reject
	Inspects/Palpates Interosseous Muscles	4	3	5	71%	Retain					
	Inspects/Palpates Thenar Muscles	4	2	5	59%	Retain					
Lower Extremities Exam	Inspects Petechiae	3	2	5	35%	Reassess	4	2	4	75%	Retain
	Inspects Purpura	3	2	4	29%	Reassess	3	2	4	69%	Retain
	Inspects/Palpates Quadriceps	5	4	5	76%	Retain					
	Inspects/Palpates Gastrocnemius	5	4	5	76%	Retain					
	Inspects/Palpates Pitting Edema	4	3	5	71%	Retain					
Functional Grip Strength Exam	Handgrip dynamometer (objective measure)	2	2	3	12%	Reassess	2	2	3	38%	Reject
	Handshake/Grip Fingers (subjective measure)	3	2	5	41%	Reassess	3	3	4	81%	Retain
Bedside Manner & Etiquette	Bilateral Inspection & Palpation	4	3	5	59%	Retain					
	Respect Patient Privacy	5	5	5	82%	Retain					
	Patient Dignity	5	4	5	77%	Retain					
	Returns Patient Arms, Legs	4	4	5	94%	Retain					
	Interviews Patient	5	4	5	94%	Retain					

* Q1—Lower Quartile, ^ Q3—Upper Quartile.

## Data Availability

The data presented in this study are available on request from the corresponding author. The data is not publicly available to protect the confidentiality and privacy of the study participants.
